# Chronic constipation due to presacral teratoma in a 36-year-old woman: a case report

**DOI:** 10.1186/1752-1947-4-23

**Published:** 2010-01-25

**Authors:** Daniel Paramythiotis, Theodossis S Papavramidis, Antonios Michalopoulos, Vassilios N Papadopoulos, Stylianos Apostolidis, Despoina Televantou, Prodromos Hytiroglou

**Affiliations:** 1 1st Propedeutic Surgical Clinic, AHEPA University Hospital, St Kyriakidi 1, Thessaloniki, 54636, Greece; 2 Laboratory of Anatomy, Aristotle University of Thessaloniki, Thessaloniki, 54124, Greece; 3 Department of Pathology, Aristotle University of Thessaloniki, Thessaloniki, 54124, Greece

## Abstract

**Introduction:**

Teratomas of the sacrococcygeal area are usually diagnosed in infancy and are rarely seen in adults.

**Case presentation:**

We report the case of a 36-year-old Greek woman experiencing chronic constipation due to a benign presacral teratoma. Imaging examinations showed a pelvic mass without evidence of malignancy. An ovoid tumour with a maximum dimension of 6 cm was surgically removed. A histologic examination revealed a mature cystic teratoma. Two years after surgery, the patient is well, with no evidence of recurrence and no constipation.

**Conclusion:**

Sacrococcygeal teratomas are rare in adults. A high index of suspicion is important in making an early diagnosis. Rectal examination and radiologic evaluation are also valuable.

## Introduction

Teratomas are tumours composed of various cell types representing more than one germ layer. Teratomas derived from germ cells occur in the gonads, whereas teratomas derived from embryonal cells are found in other locations. The sacrococcygeal area is the most frequent site of teratomas (sacrococcygeal teratomas (SCTs)) in neonates, infants, and children younger than four years. In adults, tumours at this site are very rare, occurring at a rate of between 1 in 40,000 and 63,000 [1]. SCTs have a female preponderance (3:1) [2,3]. We report the case of a 36-year-old woman who had experienced constipation for two years due to a mature presacral teratoma, which was successfully removed.

## Case presentation

**Figure 1:**
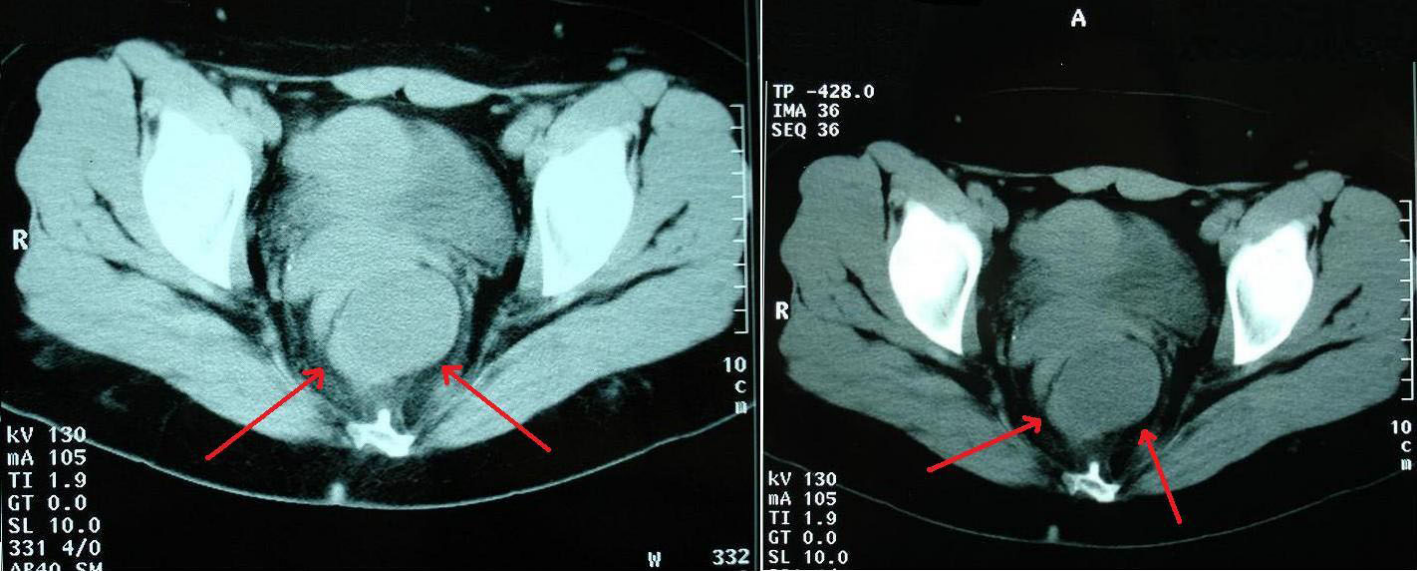
**Pelvic computer tomography shows a presacral heterogeneous mass composed of cystic component, soft-tissue elements, fatty tissue and calcifications, without evidence of bone destruction (arrows indicate the tumor)**.

**Figure 2:**
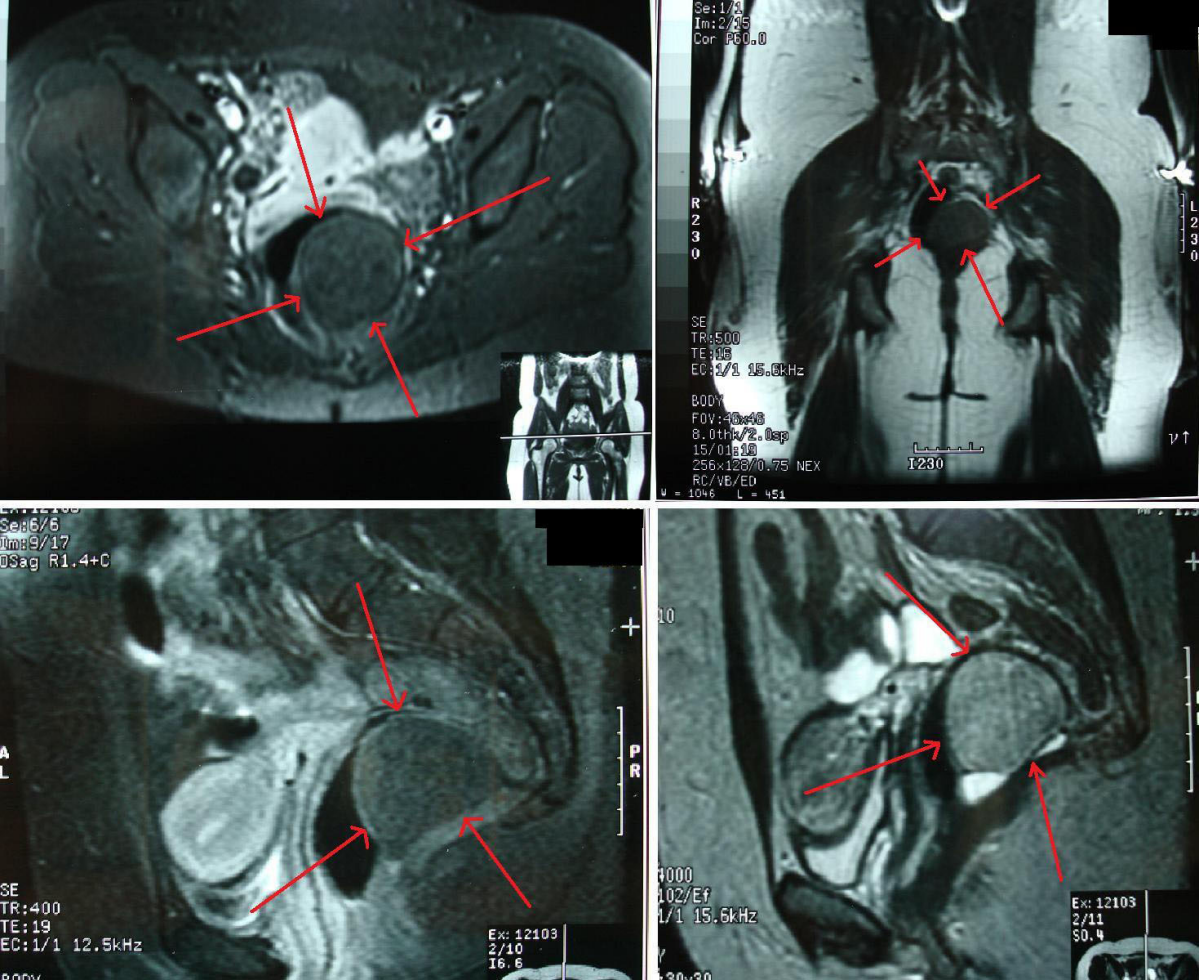
**Magnetic resonance imaging scans of the axial, coronal and midline saggital areas show a 6 × 5 × 4.5 cm mass of heterogeneous signal intensity, located anterior to the distal sacrum and coccyx (arrows indicate the tumor)**.

A 36-year-old Greek woman was admitted to our department complaining of left abdominal and pelvic pain that had developed progressively during the previous six months. She had also experienced constipation for two years, which was temporarily relieved with regular laxative treatments. Recto-sigmoidoscopy revealed extra-luminal pressure. Plain X-ray of the abdomen revealed a mass in the pelvis without extension to the sacrum. Abdominal/pelvic ultrasonography demonstrated a hypoechoic tumor in the lesser pelvis (lower sacrococcygeal region). A computer tomography (CT) scan of the pelvic area showed that the rectum and urinary bladder were displaced anteriorly by a mixed-type mass with soft tissue elements, inhomogeneous fatty tissue and calcifications, extending from the presacral area to below the coccyx (Figure 1). On magnetic resonance imaging (MRI), the mass appeared to be in close relation to the anterior surface of the sacrum. However, the lesion did not appear to be attached either to the sacrum or to the rectum (Figure 2). In addition, the MRI showed a normal lower spinal cord, a normal sacrum and no evidence of infiltrative disease in bone or adjacent tissues, such as the rectal or bladder wall, surrounding fat, or pelvic floor muscles. There was no regional lymph node enlargement.

Routine laboratory tests were within normal ranges. Human chorionic gonadotropin (hCG) and serum tumour markers, including alpha-fetoprotein (aFP), carcinoembryonic antigen (CEA) and carbohydrate antigens (CA 19-9, CA 125 and CA 15-3) were not elevated.

**Figure 3:**
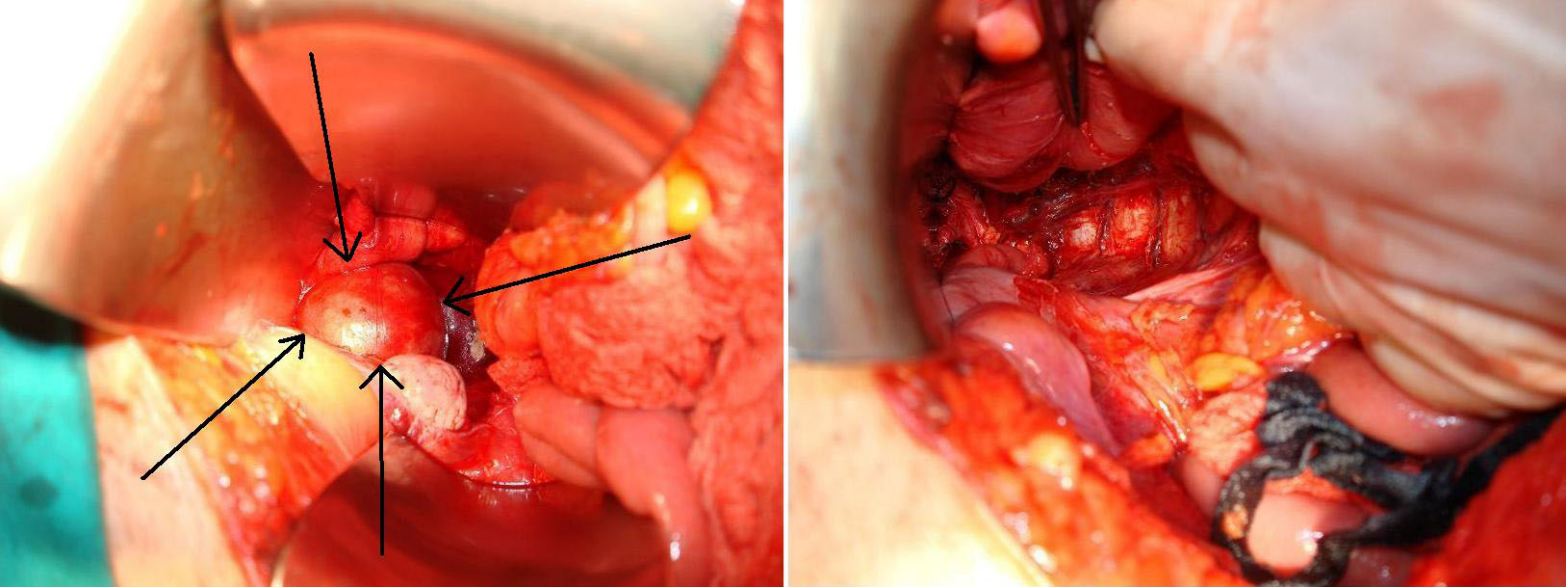
**Intra-operative macroscopic appearance of the tumour, before and after removal (arrows indicate the tumour)**.

During laparotomy, a 5 cm mass was found posterior to the rectum and anterior to the sacrum, extending between the anal sphincters (Figure 3). The tumour was mobilised easily by incising the posterior parietal peritoneum on both sides and ligating the middle sacral vessels. After the operation, the patient's recovery was uneventful, and she was discharged five days after the surgery. At follow-up two years later, the patient had had no evidence of recurrence and was free from constipation.

The surgical specimen was an ovoid cystic lesion measuring 6 × 5 × 4.5 cm. On sectioning, two cystic cavities were found, the larger with a maximum dimension of 5 cm and containing sebum, and the smaller one measuring 1.5 cm and containing mucin. On histologic examination, the larger cavity was lined with stratified keratinizing squamous epithelium and the smaller one was lined with pseudostratified mucin-producing columnar epithelium with occasional ciliated cells. The cyst wall was composed of fibro-adipose tissue, smooth muscle fiber and glandular structures with dilated lumens. A diagnosis of mature cystic teratoma was made.

## Discussion

SCTs originate either ventral or dorsal to the sacrum, and may grow posteroinferiorly into the gluteal area or anterosuperiorly into the lesser pelvis. In the ventral location, these tumours may grow to a large size as they develop into the retrorectal or presacral space. The clinical presentation of SCTs is mainly attributed to their growth and size, or to dysfunction of adjacent organs due to local pressure or infiltration [4]. When the rectal angle at the puborectalis muscle is affected, patients may present with changes in bowel function such as constipation, sensation of incomplete evacuation, narrowed stools, or incontinence [5,6]. In our case, the patient had experienced constipation for two years.

Differential diagnosis is mainly from congenital abnormalities and tumours of the presacral area, such as anterior meningocele, rectal duplication, tailgut cyst, neurogenic tumours (neurofibroma, neurilemmoma, ganglioneuroma, ependymoma, malignant peripheral nerve sheath tumour), osseous (osteogenic sarcoma, chondrosarcoma, chordoma, Ewing sarcoma), and cysts, soft tissue tumours (lymphangioma, leiomyoma, desmoid tumour, soft tissue sarcomas) as well as uterine, ovarian and metastatic tumours.

Cystic teratomas may often be complicated by inflammation and therefore may be associated with soft tissue destruction. Thus, SCTs may be misdiagnosed as high lying pararectal abscesses, fistulas with presacral extension, pilonidal cysts with abscess formation, postinjection granulomas, and osteomyelitis or tuberculosis of sacrum [6,7].

Concerning the radiological evaluation, multiple dense calcifications are present in 50% of teratomas on plain radiographs. Irregular calcifications are reported to be present in 25% of malignant teratomas [8], in comparison with 74% of the benign ones [9], so they cannot be considered a sign of benignity. The ultrasound appearance of immature teratomas is considered nonspecific, although these tumours are typically heterogeneous with partially solid lesions and usually have scattered calcifications. Malignancy is suspected in large tumours with necrotic areas, poor definition of the adjacent soft tissue planes, sacral infiltration, and certainly when locoregional lymph node and distant metastases are noted [7-9].

Thus, the preoperative diagnosis can be assisted significantly by modern imaging techniques such as CT and MRI scanning. CT scanning clearly demonstrates whether the tumour consists of cystic and/or solid components with fluid, fat, soft tissues, and calcifications. MRI can be used to evaluate the presence or absence of bone or nerve infiltration, to define accurately the tumour extent and to delineate soft tissue planes for surgical planning [8,9].

In the case of our patient, CT and MRI showed calcifications within the tumour and absence of sacral infiltration, as well as clear planes between the lesion and the adjacent organs suggesting that this was a benign tumour.

Surgery is the preferred treatment for presacral teratomas if the tumour can be completely extirpated. Posterior (trans-sacral, similar to the Kraske approach to the rectum with the patient in prone position), anterior (abdominal), or combined approaches have been used, depending on the tumour size and location [10].

The anterior approach is typically performed for high lesions without evidence of sacral involvement, as was the case in our patient. Attention must be given to the middle sacral artery, which is frequently the major supplying vessel. This approach has the advantage of providing excellent exposure of important pelvic structures, such as the iliac vessels and ureter [11].

## Conclusion

Sacrococcygeal teratomas are rare in adults. A high index of suspicion is an important factor in making an early diagnosis; rectal examination and radiologic evaluation are also valuable.

## Abbreviations

aFP: alpha-fetoprotein; CEA: carcinoembryonic antigen; CT: computer tomography; MRI: magnetic resonance imaging; SCT: sacrococcygeal teratoma.

## Consent

Written informed consent was obtained from the patient for the publication of this case report and any accompanying images. A copy of the written consent is available for review by the Editor-in-Chief of this journal.

## Competing interests

The authors declare that they have no competing interests.

## Authors' contributions

DP and TSP admitted the patient in the emergency room, analyzed and interpreted the patient's data and drafted the manuscript. AM, VNP and SA were the surgeons and the attending physicians and also revised the manuscript. DT and PH performed the histological examination of the mass. All authors read and approved the final manuscript.
